# A randomized controlled trial of the effectiveness of the mHealth program in improving the lifestyle of nursing students

**DOI:** 10.1038/s41598-024-80982-2

**Published:** 2025-03-21

**Authors:** Shaherah Yousef Andargeery, Dina S. El-Rafey

**Affiliations:** 1 Nursing Management and Education Department, College of Nursing, Princess Nourah Bint Abdulrahman University, P.O. Box 84428, 11671 Riyadh, Saudi Arabia; 2 Community, Environmental, and Occupational Medicine Department, Faculty of Medicine, Zagazig University, Zagazig, Egypt

**Keywords:** Interventional program, Lifestyle, mHealth, Nursing student, Health occupations, Medical research

## Abstract

Promoting healthy lifestyles is essential for preventing chronic diseases, yet a vast majority of university students regularly engage in unhealthy habits. Utilizing mobile smart devices for health interventions, known as mHealth, which integrate behavioral change theories with environmental interaction, offers a promising and cost-effective strategy to encourage lasting adoption of healthier habits. This study compared the effectiveness of the mHealth intervention program with a traditional face-to-face program in fostering healthy lifestyle changes. Through a randomized controlled trial involving 220 nursing students (110 in the mHealth intervention program and 110 in the traditional program), data were collected from May to December 2023 using predefined questionnaires. These questionnaires included the Global/International Physical Activity Questionnaire (GPAQ/IPAQ) for monitoring adult physical activity, a Food Frequency Questionnaire (FFQ) for dietary assessment, and a Sleep Quality Scale (SQS). Three months after the educational intervention program, lifestyle improvements were significantly more pronounced in the mHealth intervention group compared to the traditional group. The implementation of the mHealth intervention program aimed at improving lifestyle has proven to be a transformative approach in fostering positive behavioral changes among participants. The study was approved by the IRB of Zagazig Faculty of Medicine (IRB 10827/24-6-2023) and was registered at the ClinicalTrials.gov (NCT06404619, 08/05/2024).

## Introduction

A “healthy lifestyle” is a vital aspect of overall “health”, encompassing various elements such as abstaining from abuse like alcohol, tobacco, and unhealthy diets, engaging in regular physical activity either through organized means or individually as a recreational pursuit, and adhering to fundamental nutrition guidelines. A healthy lifestyle is consistently linked to good health and an active life^1^. Lifestyle interventions, encompassing beneficial alterations in dietary habits, have the potential to markedly diminish the risk of developing chronic ailments during aging^2^. The student phase is a critical period underscoring the impact of lifestyle choices. In this stage, students face various challenges, such as adjustments in lifestyle, shifts in social settings, formation of new social connections, and increasing autonomy and behavioral independence. These factors collectively influence both physical and mental well-being^3^. The shift to a new living environment, accompanied by demanding schedules, the prevalence of unhealthy food options, and the tendency to skip meals often leads to gradual changes in eating habits. In Egypt, approximately one-third of adults have high blood pressure, and nearly half of the adult male population smokes. The prevalence of obesity, high cholesterol, and physical inactivity among persons aged 15 to 69 is 28%, 14%, and 25% for men and 22%, 23%, and 49% for women^4^. In addition, in the systematic review by Memon et al.^5^, studies indicate that university students typically engage in low levels of physical activity and frequently experience sleep disturbances, both of which are independently linked to adverse health outcomes.

From an educational standpoint, the university years are often viewed as the final opportunity to provide many students with essential nutritional education^6^. The diets of students worldwide are frequently characterized as unhealthy, marked by low fruit and vegetable intake, irregular meal patterns, and a high reliance on fast food. This is especially concerning, as dietary habits formed during this phase can significantly impact long-term health outcomes^7^. Furthermore, behaviors adopted by students during university can have broader community implications, as young adults often assume influential societal roles—such as physicians, police officers, attorneys, nurses, and health ministers. Public health is particularly interested in university students’ health and lifestyle choices because of their capacity to make decisions with substantial behavioral patterns and attitudes^8^.

Leveraging mobile health (mHealth) tools to empower populations represents a crucial step in enhancing overall health, especially considering that chronic illnesses, including cardiovascular diseases, respiratory diseases, cancer, and diabetes, account for a significant portion of global mortality^9^. The adoption of mHealth technology offers a promising approach to the promotion of health behavior change among university students by enabling personalized and convenient interventions. Students may track their progress and make the required corrections thanks to mHealth’s real-time monitoring and feedback capabilities. Additionally, it fosters social support through social networks and messaging apps, and provides a cost-effective alternative to traditional in-person interventions, reducing the need for extensive resources^10^

Nurses are at the forefront of advancing the science of mHealth, from creating mobile applications for post-traumatic stress disorder management to employing evidence-supported text messages for sustaining weight loss, these initiatives span a spectrum of contributions^11^. They can drive the transition from hospital-centered healthcare to personalized and community-oriented care, guiding clinicians and researchers in formulating evidence-driven mHealth frameworks^12^. Nurses and nursing students engage directly with patients, providing care and advocating on their behalf. They are supposed to act as good behavior role models for patients and their families. As a result, nurses and nursing students must embrace a healthy lifestyle and stay aware so they can actively support it throughout their employment^13^. The study’s objectives were to compare the effects of in-person education programs and mHealth intervention programs on promoting healthy lifestyles and to assess the impact of the former on the latter.

## Methods

### Study design and population

The present research was a randomized open-label controlled trial (interventional study) among students of the Faculty of Nursing, Zagazig University. The study received approval from the dean of the Faculty of Nursing and the Institutional Research Review Board (IRB) of the Zagazig Faculty of Medicine (IRB 10827/24-6-2023). Every subject gave their informed consent to participate in the study, and all experiments were carried out in compliance with applicable laws and regulations, protecting people from injury or unexpected consequences. Inclusion criteria were age between 19 and 24 years both male and female, BMI ≥ 18.5, performing physical activity (PA), and being prepared to use several social media platforms using a smartphone. However, we did not include students who are on a diet or have previously followed one, have a history of chronic illnesses such as diabetes mellitus, hypothyroidism, or other endocrine disorders, or have used medications that can lead to weight increase, such as antipsychotics or antidepressants, epileptic medicines, and steroids or students with mental or psychological disorders. Data was collected from May to December 2023.

### Sample size and technique

The mean health behavior before and after the intervention was deemed as 46.15 ± 4.57 and 48.08 ± 5.59, respectively, indicating the impact of the health promotion intervention on nurses’ healthy lifestyle^14^ with a confidence level of 95% and 80% study power. So, the sample size calculated by the Open Epi program was 220 students to be taken from 1st to 4th grade by simple random technique with proportional allocation, and the enrollment in intervention groups was done using block randomization by computer software. The sample was classified randomly according to intervention type by block randomization using a sealed Envelope website^15^ into 22 blocks; each block size 10 list length with an allocation ratio of 1:1.

### Study tools

The study was carried out through three stages:

#### Preintervention

##### First tool

All participants were met face to face, welcomed then filled out the questionnaire which consisted of socio-demographic characteristics for the participant students e.g., age, sex, residence, family income, father education, mother education, father occupation, and mother occupation^16^*.*

**Data on Global/International Physical Activity Questionnaire (GPAQ/IPAQ)** The World Health Organization (WHO) created this survey to track persons’ levels of physical exercise. It assesses activity across different domains: work, transportation, and recreational time. The questionnaire has been validated for use in Arabic and self-administration^17^.

***Physical activity time and levels*** were calculated as follows:

- Multiplying the number of days in each category by the average daily duration yields the total weekly time spent on physical activity as well as the time spent in each area. Metabolic equivalents (MET) are then multiplied by the weekly minutes in each category. The computation of MET scores involves multiplying the moderate activity **level by a factor of 4, while the severe activity level is multiplied by 8.
- METs (Metabolic Equivalents) are used in the processing of GPAQ (Global Physical Activity Questionnaire) data and act as a standard metric for measuring the intensity of physical activities.

The ratio of a person’s resting metabolic rate to their active metabolic rate is known as MET. One MET is equal to one kcal/kg/hour, which is the energy expenditure of sitting quietly. According to the guidelines used for GPAQ data analysis, calorie expenditure increases fourfold during moderate activity and eightfold during strenuous activity as compared to sedentary behavior.

Accordingly, 4 METs are allocated to moderate activity and 8 METs to intense activity when calculating an individual’s total energy expenditure using GPAQ data.

Using GPAQ data, the following MET values are used to determine an individual’s total energy expenditure:

The total activity subdomain score is determined by aggregating the MET scores from both moderate and severe activity levels.

The overall GPAQ score is derived by subtracting the cumulative score of sedentary subdomains from the total activity subdomain score.

- Low “Inactive” means less than 600 MET minutes per week; moderate means between 600 and 1500 MET minutes per week; high means more than 1500 MET minutes per week.
- **Dietary assessment using Food Frequency Questionnaire (FFQ)**^18^. This includes 140 food products divided into 27 categories. Following the removal of components that contributed insignificantly to food commonality or between-person variance, these meal items were streamlined. Subsequent investigation revealed that the streamlined survey had acceptable test–retest reliability, known-group validity, predictive validity, concurrent validity, convergent validity, and content validity. When combined with calibration factors, it is both valid and dependable. An effective tool for nutritional assessment is this FFQ^19,20^.
- The three Likert scales for these 27 categories are "usually eat," "sometimes eat," and "rarely eat." The questionnaire is subdivided into 3 equal columns (A, B, and C) with the same number of choices. Each choice gives one point. For scoring we multiplied, the total checks in column A by 1, the total checks in column B by 2, and the total checks in column C by 3 and wrote the total score by adding all scores together. If your score is:
  - 27–45: bad healthy choices.
  - 46–63: fair healthy choices
  - 64–81: good healthy choices

**Sleep quality scale**^21^; With a Cronbach’s alpha coefficient of 0.90, the sleep quality scale (SQS) score homogeneity was high. The SQS has been validated as a valid and highly reliable tool for evaluating sleep quality^22^ and is used to determine the quality of sleep during the past month. It had four Likert scales (rarely, sometimes, often, and almost usually) and 28 questions. Each item on a Likert-type scale, which typically ranges from 1 to 4, is rated by participants in a sleep quality exam.

### Examples

Occasionally: 1–2 times per week Frequently: 3–5 times per week Seldom: None or 1–3 times per month.

Almost always: six to seven times every week.

A higher score may indicate more frequent sleep disturbances in relevant items. The total score was derived by summing the individual item scores, serving as an indicator of overall sleep quality, with higher total scores reflecting poorer sleep quality. Total scores can range from 0 to 84, Higher ratings indicate more severe sleep issues, which were taken into account:

Excellent sleep ≤ 28, fair 29– ≤ 56, and terrible > 56.

### The second tool includes

I-Anthropometric measurements concerning weight, fat percentage, and height were measured. Then BMI was calculated (weight (kg)/height (m^2^). Each student was categorized according to their BMI according to WHO^23^ into:

$${\text{Underweight}}\;{\text{less}}\;{\text{than}}\;18.5\;{\text{kg/m}}^{2}$$

$${\text{normal}}\;\left( {18.5{-}24.9\;{\text{kg}}/{\text{m}}^{2} } \right)$$

$${\text{Over}}\;{\text{weight}}\;{\text{of}}\;{25}\;{\text{to}}\;{29}.{9}\;{\text{kg}}/{\text{m}}^{{2}}$$

$${\text{Obeses }}( \ge 30 {\text{kg}}/{\text{m}}^{{2}} )$$

II-Assessment of blood pressure.

**Blood Pressure Levels were classified according to The 2023 European Society of Hypertension (ESH)** ^24^

The normal blood pressure is less than 120/80 mmHg.

Prehypertension (at risk for high blood pressure): 120/80 to 140/90.

Hypertension (high blood pressure): more than 140/90 mmHg^25^.

Steps

- 30 min before taking your blood pressure, avoid eating or drinking anything.
- Before reading, empty your bladder.
- Before you read, spend at least five minutes in a cozy chair with a supported back.
- Keep your legs uncrossed and place both feet flat on the ground.
- Place the rest arm’s cuff at chest height on a table.
- Verify that the blood pressure cuff fits comfortably without being excessively tight. Instead of covering clothing, the cuff should be placed against bare flesh.
- When having your blood pressure checked, avoid talking.

III-Laboratory investigation:

Participants were asked to measure random blood sugar and lipid profiles before and three months after intervention at the Zagazig University Hospital laboratory.

- A lipid panel was used to measure the lipid profile, and the results were categorized into total cholesterol, low-density lipoproteins (LDL), high-density lipoproteins (HDL), and very low-density lipoproteins (VLDL) based on their density according to the **ACC/AHA Updates Guideline (2019).**

**Reference range for total cholesterol:**

- 200 mg per deciliter (mg/dL) or less is normal.
- 201 to 239 mg/dL is borderline.
- 240 mg/dL or more is high.

**For HDL (“good cholesterol”), more is better:**

- 60 mg/dL or higher is good – it protects against heart disease.
- 40 to 59 mg/dL is OK.
- Less than 40 mg/dL is low, raising your chance of heart disease

**For LDL (“bad cholesterol”), lower is better:**

- It is preferable to have less than 100 mg/dL.
- Depending on your health, 100–129 mg/dL can be considered good.
- The range of 130 to 159 mg/dL is borderline elevated.
- The range of 160–189 mg/dL is high.
-  > 190 mg/dL is considered extremely high.

#### Intervention stage

The students had been randomly divided into two groups according to the type of intervention program into **Group (A)** received a healthy lifestyle through the mHealth education program. One session was conducted every two weeks for this group and each session lasted for an hour Z, on Zoom meetings for three months. The mHealth education material was delivered through a PowerPoint presentation containing educational videos and pictures and the material was sent to them at the end of each session. The number of coaching calls completed and text message responses were used to gauge daily participation in the intervention. Participants in the intervention were prompted to respond “Done” by email or social media.

**Group (B)** received a healthy lifestyle through a face-to-face program in a classroom in the Faculty of Nursing at Zagazig University. One session was conducted every two weeks for this group and each session lasted for an hour at their convenience place for ***three*** months, through face-to-face classroom-based teaching. There were PowerPoint presentations, small group discussions, and the exhibition of educational messages, posters, and handouts for every student in the group.

### Message

The message was given to both group (A, B), message discussing the importance of life quality and the tenets of positive psychology are emphasized in the message. It defines quality of life as a multifaceted notion and lists the necessary steps for changing one’s lifestyle, including introspection, habit modification, and dedication to a comprehensive change. The importance of exercise, stress reduction, and maximizing the length and quality of sleep are important elements. Specific guidelines emphasize the importance of hydration, structured meal planning, and adopting a well-balanced diet with mindful portion control to stabilize blood sugar levels and supply necessary nutrients. Additionally, the message discusses fitness foods that support digestion and overall health and introduces the Healthy Eating Plate as a visual guide for balanced eating.

#### Post-intervention phase

The primary outcome was the improvement in the students’ scores in physical activity, dietary assessment, and sleep quality which was assessed by asking the students to refill the questionnaires; the identical dietary evaluation scale, the GPAQ/IPAQ, and the same sleep quality scale.

The secondary outcome was an improvement in anthropometric measurement and laboratory findings which was assessed by remeasurement of the following BMI, blood pressure, blood sugar, and lipid profile.

### Statistical analysis

SPSS 27.0 software was used for data collection, tabulation, and statistical analysis. To determine whether the data distribution was normal, the Shapiro–Wilk test was used. Frequencies and relative percentages were used to display categorical data. When necessary, the Chi-square test (χ2) was used to determine how qualitative variables differed from one another. The range and mean ± SD (standard deviation) were used to report quantitative data. The paired t-test was used to compare pre- and post-intervention data within the same group (paired data), whereas the independent t-test was used to assess differences between quantitative variables in different groups for parametric data.

## Results

**Fig. 1:**
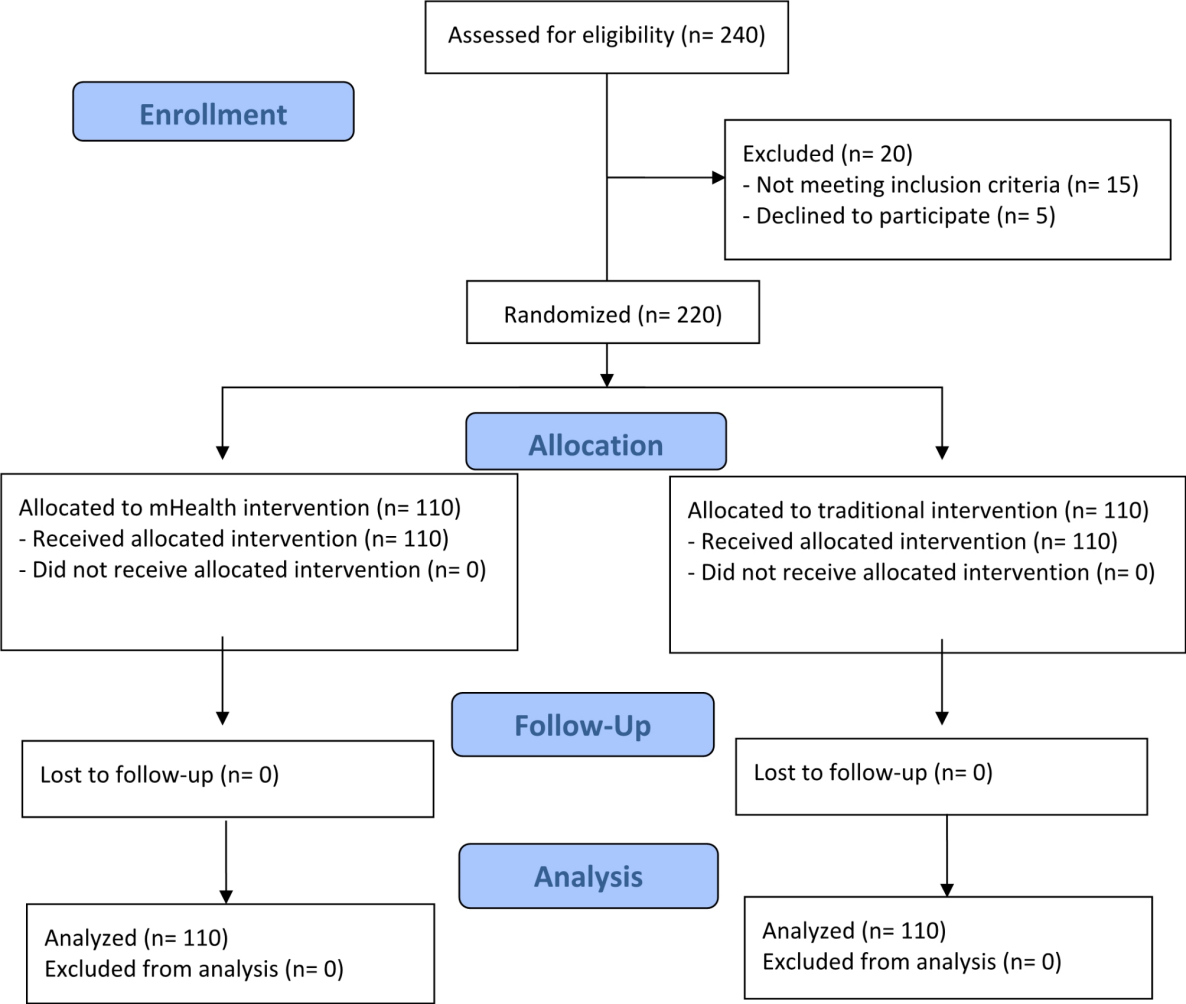
CONSORT flow diagram of the study.

In total, 220 participants were included in a randomized interventional study, 110 participants were in the mHealth interventional program, and 110 participants were in the traditional (face-to-face interventional) program (Fig. 1). The mean age of participants in the mHealth program and traditional program were (20.5 ± 2.41, and 19.97 ± 2.61) respectively. Regarding gender, 40% were male, 60% were female in the mHealth program 46.3% were male and 53.7% were female in the traditional program. About two-thirds of participants were from rural areas in the mHealth group and 59.1% were from rural areas in the other group. Most of the studied groups were with moderate socioeconomic status (45.4%, 47.3%) in the mHealth group and traditional group respectively (Table 1).

**Table 1:** Difference between the two studied groups as regard the sociodemographic characteristics.

Sociodemographic characteristics	Group A		Group B		P value
	(mHealth)		(Traditional face-to-face intervention)		
	(N = 110)		(N = 110)		
Age (years)					0.11 €
Mean ± SD	20.5 ± 2.41		19.97 ± 2.61		
Range	(18–24)		(18–24)		
Gender	N	%	N	%	0.34 #
Male	44	40%	51	46.3%	
Female	66	60%	59	53.7%	
Residence					0.26 #
Rural	73	66.4	65	59.1	
Urban	37	33.6	45	40.9	
Socioeconomic level					0.89 #
Low	32	29.1%	33	30%	
Middle	50	45.4%	52	47.3%	
High	28	25.4%	25	22.7%	

€: Independent t-test; #: Chi-square test; N: number; SD: standard deviation.

**Fig. 2:**
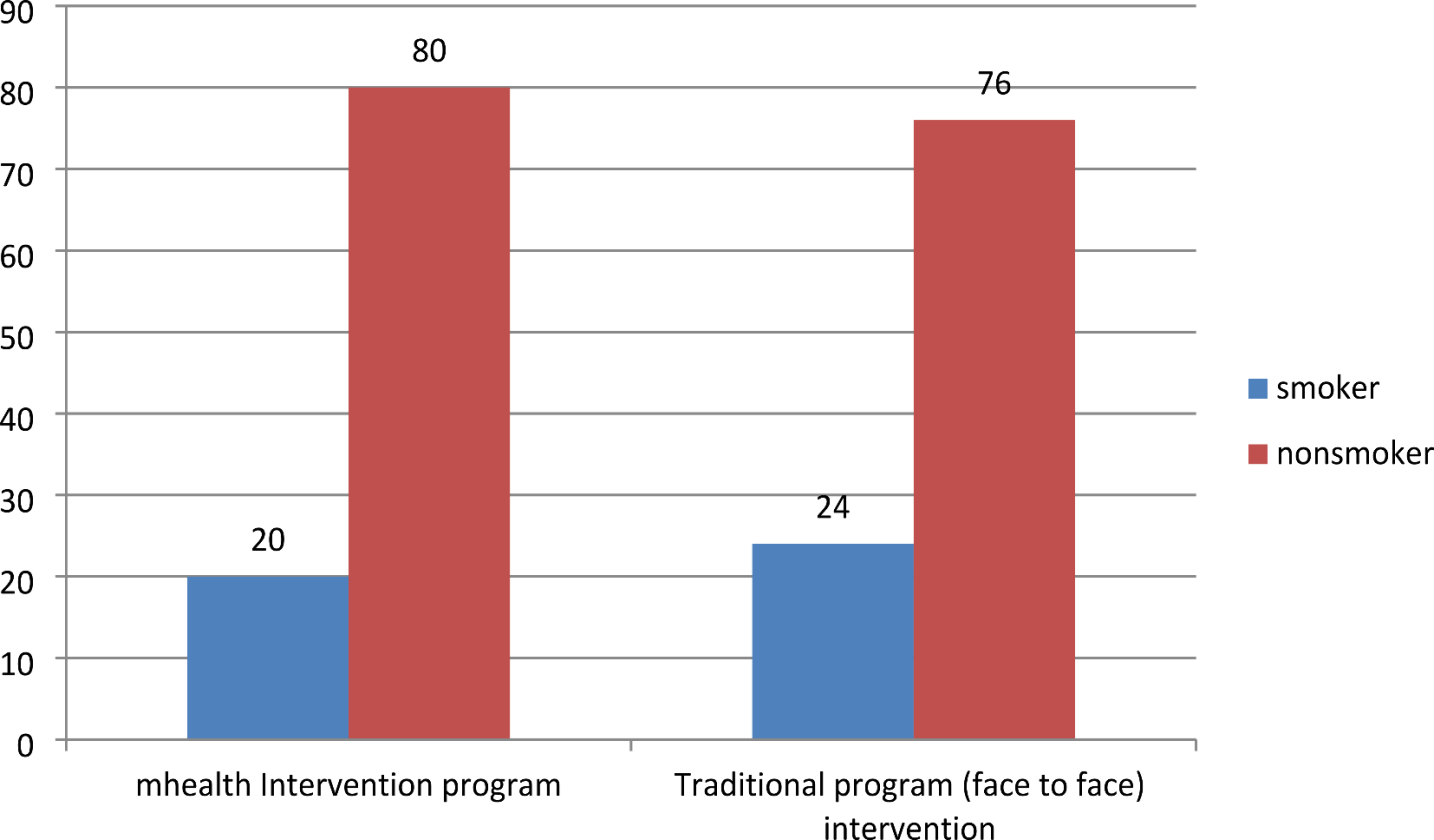
Smoking status among two studied group.

**Fig. 3:**
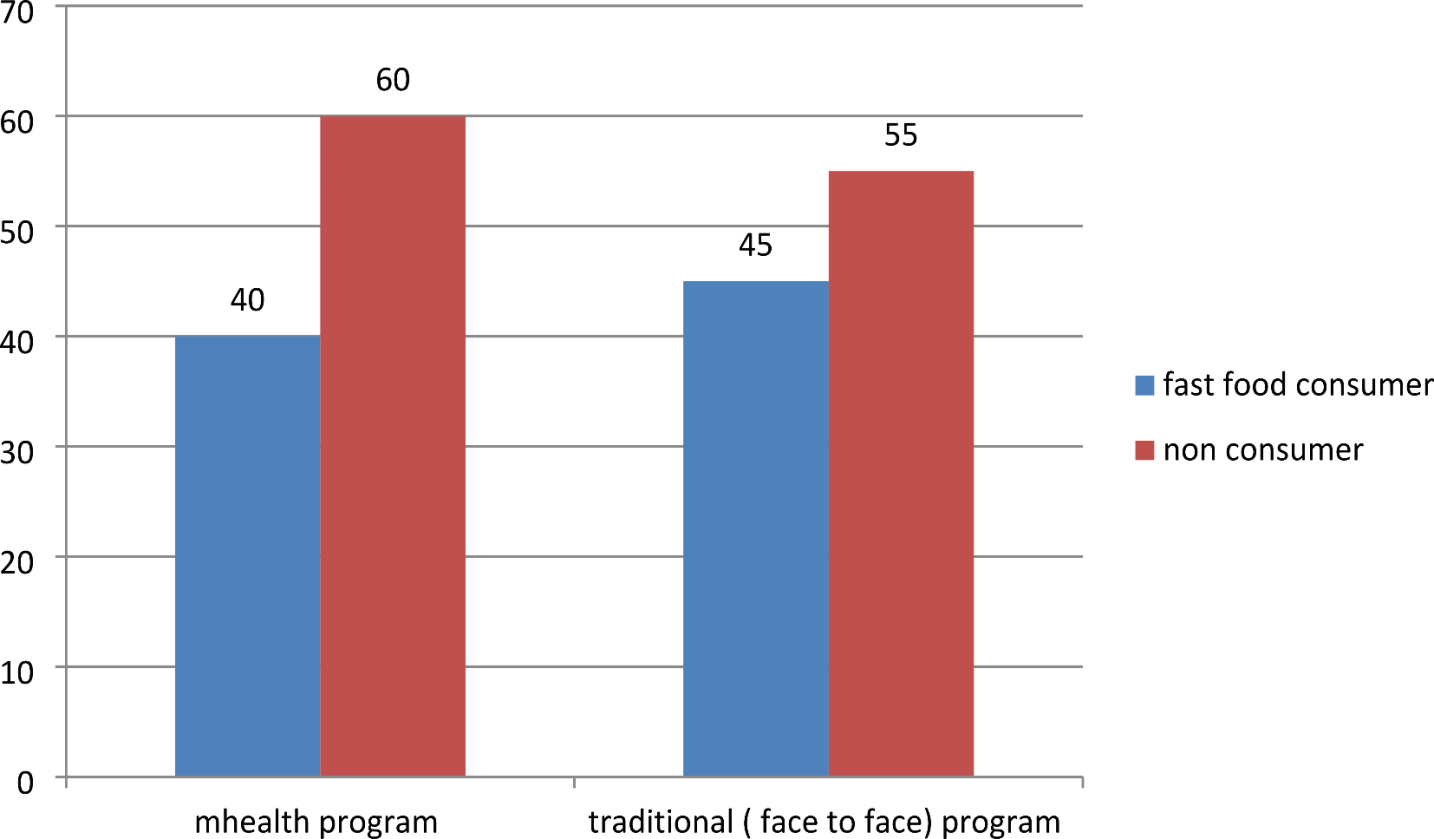
Prevalence of fast food consumption habits among the studied students.

**Fig. 4:**
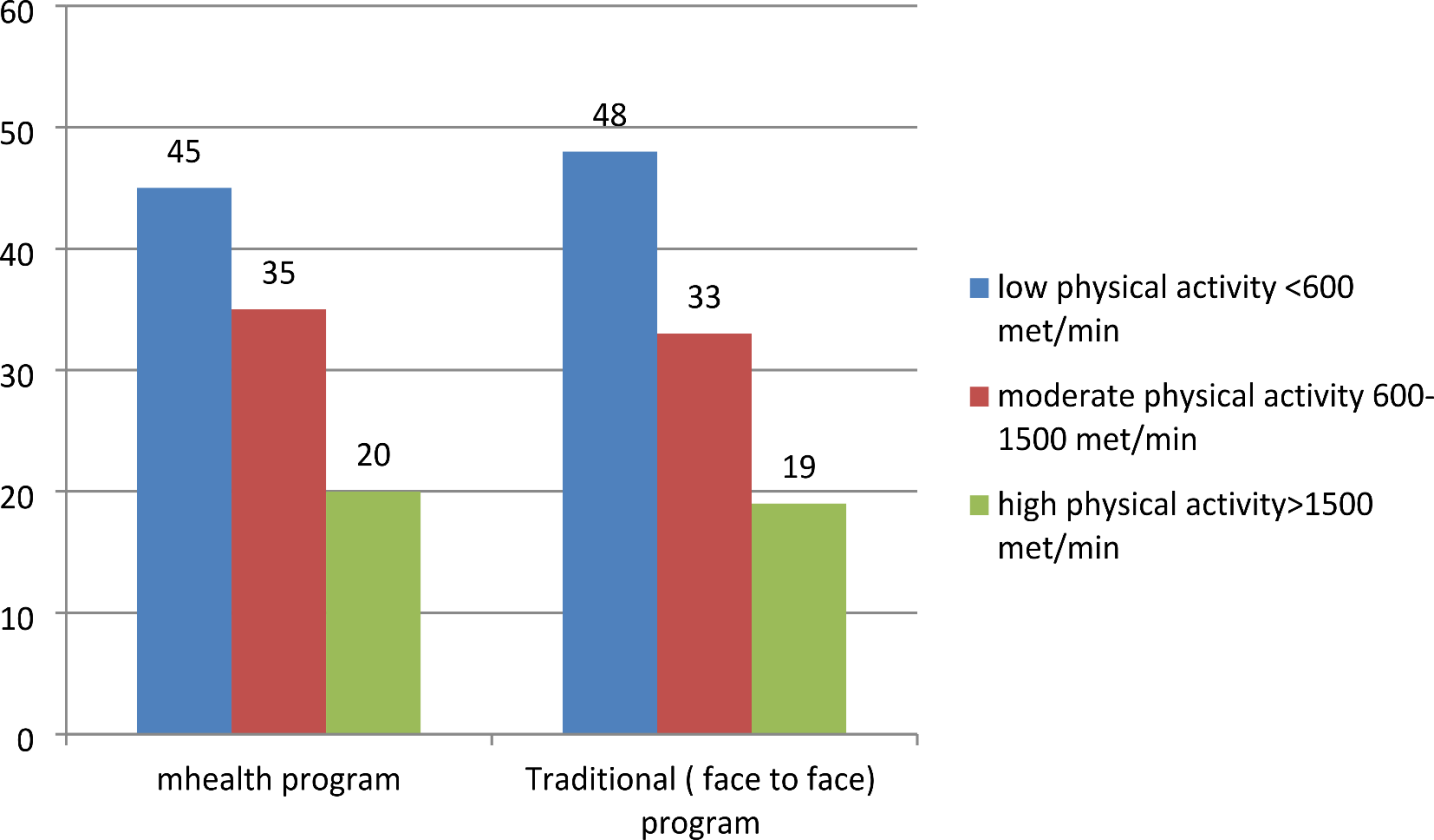
Assessment Physical Activity (IPAQ) among the studied students.

**Fig. 5:**
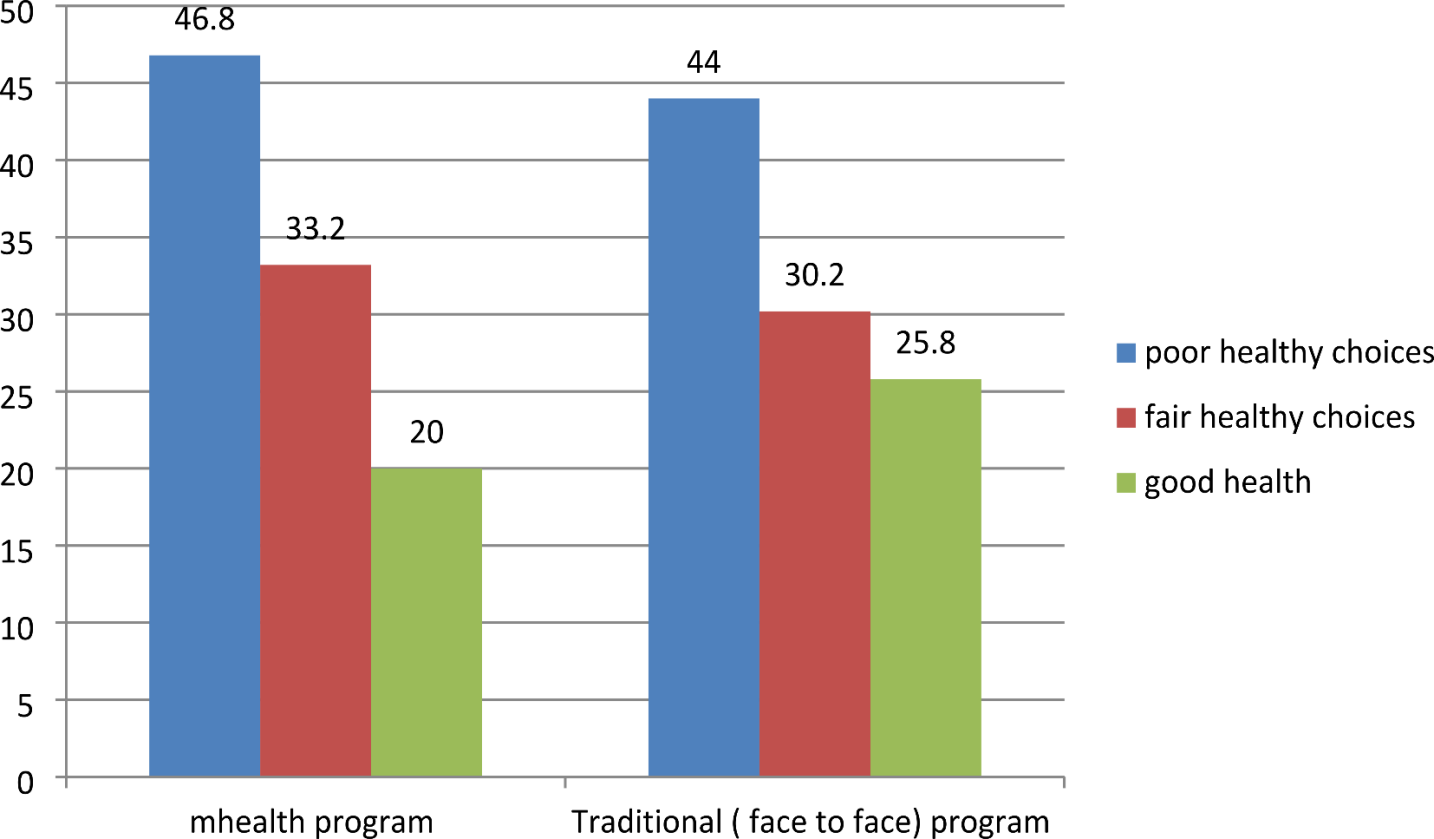
Assessment of studied group as regard dietary behavior.

**Fig. 6:**
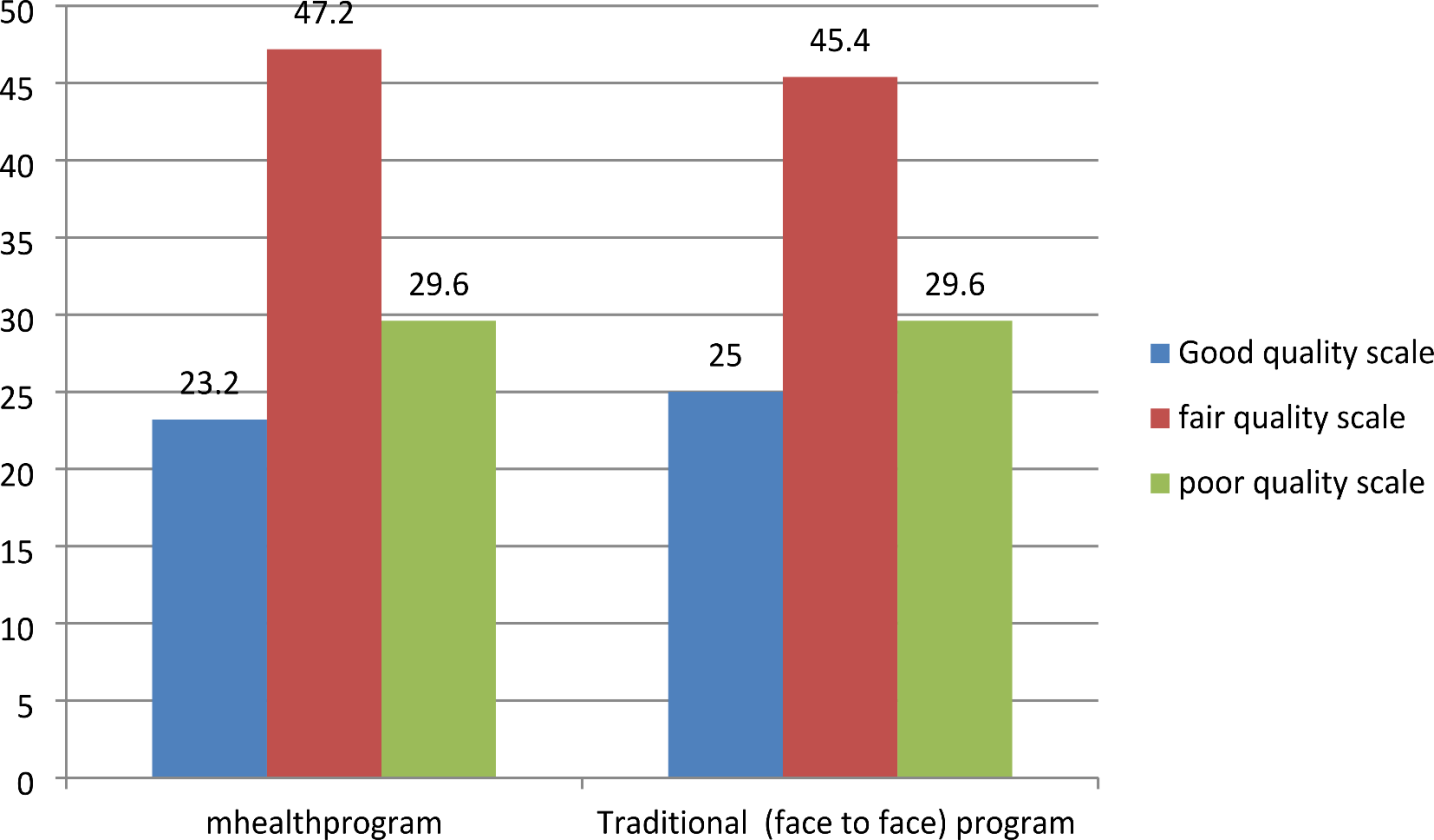
Assessment of studied group as regard Sleep Quality Scale.

Regarding the pattern of lifestyle among the studied group, we found about (20%, and 24%) were smokers in the mHealth and traditional groups respectively (Fig. 2). Regarding fast food consumption, 40% of participants in the mHealth group were fast food consumers versus 45% of the traditional group (Fig. 3). Most of the participants practiced low physical activity less than 600 met/min (45%, and 48%) in the mHealth group and traditional group respectively (Fig. 4), 46.8%, and 44% of participants in the mHealth group and traditional group respectively with poor healthy choices (Fig. 5) and 47.2% and 45.4% with fair sleep quality (Fig. 6).

There is a statistically significant improvement in physical level (IPAQ), food frequency questionnaire (FFQ), and sleep quality scale (SQS) post-intervention compared to pre-intervention in mHealth and the traditional groups. Also, there was a statistically significant improvement in mHealth versus the traditional group regarding (physical activity (IPAQ), food frequency questionnaire, and sleep quality scale (SQS) (Table 2).

**Table 2:** Comparing lifestyle (physical activity, Food Frequency Questionnaire and Sleep Quality Scale among the two studied groups.

	Group A	Group B	P value
	(mHealth)	(Traditional face-to-face intervention)	
	(N = 110)	(N = 110)	
	Mean ± SD	Mean ± SD	
	Range	Range	
Global/International Physical Activity Questionnaire (MET/Min)			
Pre	450 ± 38	444 ± 57	0.35 €
	(275–888)	(272–860)	
Post	1895 ± 214	1837 ± 188	0.03* €
	(1369–3449)	(943–3406)	
P value	< 0.001*#	< 0.001*#	
Food frequency questionnaire			
Pre	41.17 ± 13.45	40.83 ± 11.93	0.84 €
	(11–62)	(20–60)	
Post	58.17 ± 14.73	53.73 ± 10.92	0.011*€
	(23–81)	(25–80)	
P value	< 0.001* #	< 0.001* #	
Sleep quality scale			
Pre	38.47 ± 14.45	36.63 ± 13.04	0.32 €
	(7–66)	(6–61)	
Post	28.53 ± 9.79	31.43 ± 9.91	0.009* €
	(3–56)	(16–51)	
P value	0.019 *#	0.012*#	

€: Independent t test; #: Paired t-test; *: statistically significant; MET/Min: Daily Metabolic Equivalent Minutes; N: number; SD: standard deviation.

Table 3 indicates no statistically significant difference between the two groups’ BMIs or between their pre and post-intervention BMIs; however, the mHealth group’s and the traditional group’s systolic blood pressure improved statistically significantly after the intervention compared to before (p-value < 0.001, < 0.001 ) respectively and in diastolic blood pressure improvement more in mHealth (p-value = 0.002), there was statistically significant improvement post-intervention in mHealth group versus traditional group (p-value = 0.003) regarding systolic blood pressure.

**Table 3:** Comparing clinical measurements pre- and post-intervention between the two studied groups.

Clinical measurements	Group A	Group B	P value
	(mHealth)	(Traditional face-to-face intervention)	
	(N = 110)	(N = 110)	
	Mean ± SD	Mean ± SD	
	Range	Range	
Weight (kg)			
Pre	68.29 ± 13.3	67.84 ± 11.6	0.78 €
Post	66.17 ± 11.3	65.12 ± 11.2	0.48 €
P value	0.20#	0.078#	
Fat (%)			
Pre	23.3 ± 8.4	23.12 ± 9.4	0.88 €
Post	22.3 ± 6.4	22.8 ± 8.5	0.62 €
P value	0.32#	0.79#	
Body mass index (kg/m^2^)			
Pre	25.03 ± 2.41	25.28 ± 2.67	0.46€
	(20.7–31)	(20.4–31.8)	
Post	24.73 ± 4.16	25.1 ± 4.25	0.51€
	(18–30.9)	(18–32.5)	
P value	0.728#	0.845#	
Systolic blood pressure (mmHg)			
Pre	124.83 ± 3.97	124.97 ± 3.21	0.14€
	(118–133)	(119–133)	
Post	120.47 ± 4.59	121.7 ± 3.76	0.03*€
	(110–127)	(113–130)	
P value	< 0.001#	0.001#	
Diastolic blood pressure (mmHg)			
Pre	81.3 ± 3.5	80.4 ± 3.9	0.073 €
	(78–86)	(75–88)	
Post	79.63 ± 2.72	79.07 ± 2.79	0.133 €
	(75–85)	(75–86)	
P value	0.002#*	0.081#	

€: Independent t-test; #: Paired t-test; *: statistically significant; N: number; SD: standard deviation.

Regarding lipid profile, there was a statistically significant decrease in (TC (< 0.001, < 0.001), LDL (< 0.001, < 0.001), HDL (0.04, 0.005), and RBS (< 0.001, < 0.001) post-intervention compared to pre-intervention in the mHealth group and traditional group respectively. Also, in comparing the mHealth group versus the traditional group there was a statistically significant improvement in mHealth more than the traditional group in TC and LDL (p-value = 0.04, 0.05, respectively) (Table 4).

**Table 4:** Comparing lipid profile pre and post intervention among the studied groups.

Lipid profile	Group A	Group B	P value
	(mHealth)	(Traditional face-to-face intervention)	
	(N = 110)	(N = 110)	
	Mean ± SD	Mean ± SD	
	Range	Range	
Total cholesterol (mg/dl)			
Pre	189.3 ± 31.31	186.1 ± 33.55	0.46 €
	(124–253)	(121–271)	
Post	146.4 ± 20.63	152.4 ± 24.83	0.043* €
	(103–206)	(99–213)	
P value	< 0.001*#	< 0.001*#	
Low density lipoprotein (mg/dl)			
Pre	126.17 ± 24.64	125.23 ± 35.77	0.82 €
	(65–170)	(50–192)	
Post	87.23 ± 20.3	95.53 ± 22.47	0.004* €
	(46–122)	(57–161)	
P value	< 0.001*#	< 0.001*#	
High density lipoprotein (mg/dl)			
Pre	44.03 ± 5.95	43.9 ± 3.76	0.13 €
	(24–56)	(35–53)	
Post	46.57 ± 3.75	45.83 ± 5.21	0.22 €
	(39–64)	(36–60)	
P value	0.04* #	0.005*#	
Random blood sugar (mg/dl)			
Pre	116 ± 15.22	114.5 ± 17.36	0.49 €
	(85–147)	(82–143)	
Post	96.17 ± 8.35	95.5 ± 9.62	0.58 €
	(82–121)	(81–117)	
P value	< 0.001*#	< 0.001*#	

€: Independent t-test; #: Paired t-test; *: statistically significant; N: number; SD: standard deviation.

## Discussion

The potential of digital communication tools in enhancing health literacy and improving health outcomes for both patients and healthcare providers is significant. Research demonstrates the effectiveness of various digital communication tools including mobile health apps, in promoting health literacy and digital literacy. These tools have been shown to facilitate patient education, self-management, and clinical decision-making^26–28^.

Many health-related behaviors and patterns of behavior often take root during adolescence and young adulthood, persisting well into adulthood. The period coinciding with college or university attendance holds particular significance for the cultivation of healthy lifestyle habits, carrying potentially significant implications for long-term health outcomes^29^.

According to our research, participants in the traditional program were 19.97 years old on average, but those in the mHealth program were 20.5 years old. Regarding the distribution of genders, 40% of participants in the mHealth program were male and 60% were female, compared to 46.3% male and 53.7% female in the traditional program. Approximately two-thirds of the participants in the mHealth group hailed from rural areas, while 59.1% came from rural areas in the traditional group. Moreover, the majority of both groups belonged to a moderate socioeconomic status, with percentages of 45.4% and 47.3% in the mHealth and traditional groups respectively.

In examining the lifestyle patterns of the participants, we observed that approximately 24% of individual in the traditional group, and 20% of individual in the mHealth group smoked whether they smoked cigarettes or used waterpipes, this finding is in agreement with Al Ali and Khazaaleh^30^. According to the WHO’s findings, 16% of students were found to be cigarette smokers, while 17% were waterpipe smokers. Consistent with earlier research, waterpipe smoking prevalence exceeded that of cigarette smoking among students. This trend could be linked to the perception of waterpipe smoking as less harmful than cigarette smoking, coupled with its social acceptability and expected behavior^31^.

In terms of fast food consumption, 40% of individuals in the mHealth group were fast food consumers, while the other group had a slightly higher rate of 45%. Additionally, 46.8% and 44% of participants in the mHealth and traditional groups respectively exhibited poor dietary choices, this is in the same line with Al Ali and Khazaaleh^30^ who revealed that over half of the students had consumed unhealthy items within the past 24 h, often prioritizing fast food over fruits and vegetables. These findings closely resembled those of Alzahrani et al.^32^ who reported that around two-thirds of students frequented fast-food restaurants occasionally, with 28.0% doing so often, and 6.1% abstaining altogether. Similarly, national surveys among Jordanian^33^ and Lebanese students^34^ corroborated these results. Fast food consumption among students can be attributed to its ready availability in and around university campuses, where healthier food options may be limited, therefore raising the chance of gaining weight and developing chronic illnesses.

Our findings indicate that a significant proportion of participants exhibited low levels of physical activity, characterized as being under 600 MET minutes. Around 48% of people in the traditional group and 45% of those in the mHealth group showed this trend. These findings align with research by Verma et al.^35^ that used the International Physical Activity Questionnaire Long Form and discovered that 14.5% of all students had low levels of physical activity, with 14.2% falling into the moderate category and thus, indicating that 28.7% of participants had low physical activity levels. Similarly, Mahfouz et al.^36^ reported that over half of their study participants (62.7%) engaged in low physical activity. Notably, college students exhibit a high rate of physical inactivity, with 52.2% reported as inactive, and 34.4% categorized as overweight or at risk of becoming overweight/obese later in life^37^. These findings align with Awadalla et al.'s observations that 48% are of low levels of physical exercise among Saudi Arabian students at King Khalid University^38^. The prevalence of low physical activity in this demographic can be attributed to the limited time students have available for regular participation in physical activity programs. However, in contrast, Ali and Khazaaleha^30^ found that 58% of the undergraduate students surveyed regularly engaged in sports activities.

It is essential to assess how well different mHealth tactics work to enhance risk factor management and general lifestyle. Our research showed that both the mHealth and traditional groups experienced significant improvements in physical activity after the intervention (p values < 0.001, < 0.001), and that there was a statistically significant difference between the two groups, with the mHealth group showing greater improvement. These findings in harmony with Al-Nawaiseh et al.^39^ who found that the randomized controlled trial demonstrates that a 12-week mHealth intervention utilizing a mobile health application led to a significant enhancement in physical activity (measured by step counts) and reduction in body weight among college students. Our findings align with those of Safran Naimark et al., who observed a 26.9% increase in physical activity (step counts) following a pedometer-based mHealth intervention compared to baseline levels. This evidence serves as a foundation for designing an optimal mHealth intervention aimed at maximizing improvements in physical activity engagement, if sustained, is anticipated to yield various health benefits, including reduced risks of obesity, heart disease, and type 2 diabetes mellitus (T2DM).

In addition, our results declared that significant improvement in (FFQ) post-intervention in both groups (p < 0.001, < 0.001) respectively, and sleep quality scale (SQS) (p-value 0.019 and 0.012). Additionally, mHealth showed a statistically significant improvement over the traditional group in terms of (IPAQ), (FFQ) and (SQS) (p-value 0.03, 0.01, 0.009) respectively. Our results, consistent with Zhang et al.^18^ demonstrated that lifestyle interventions greatly enhanced the four characteristics of behaviors’ self-efficacy, as well as the individual dimensions and general health-promoting behaviors (nutrition, psychological well-being, stress management, and physical activity). Also, our results were similar to the characteristics of another patient-centered transitional care program run by nurses, which demonstrated significant effects on self-efficacy^40^. Various behavioral theories propose similar strategies for enhancing self-efficacy, including social persuasion, modeling, and mastery of experience^41,42^,

There was a slight improvement in BMI post-intervention but still statistically insignificant, this is in the same line with Al-Nawaiseh et al.^39^ who found that there is no significant improvement post-intervention and between the intervention and control group regarding weight, fat (%) and BMI. Also, Cruz-Cobo et al.^43^ revealed the implementation of mHealth interventions did not result in a notable decrease in patients’ BMI and waist circumference, unlike the findings reported by Chen et al.^44^, which showed that BMI significantly improved as a result of mHealth interventions.

On the contrary, the mHealth group and the traditional group showed statistically significant improvements in their systolic blood pressure (p-values < 0.001, < 0.001) and diastolic blood pressure (p values < 0.002) after the intervention, respectively, compared to the pre-intervention (p-value 0.003) regarding systolic blood pressure. In consistence with the previous findings, David et al.^45^ found that a lifestyle program enhanced with mHealth interventions featuring blood pressure monitoring and supportive text messages resulted in statistically significant and clinically meaningful improvements in adherence to achieving at least four lifestyle goals, compared to standard clinical treatment (UCT) alone. Chen et al.^46^, conducted a study in China and found that eHealth interventions significantly impacted systolic blood pressure (SBP), with six studies reporting a standardized mean difference (SMD) of -0.35 (95% CI: -0.66 to -0.04, p = 0.03). Similarly, research by Zha et al., Haas et al., and Nolan et al.^47–49^ demonstrated that eHealth interventions effectively improved SBP levels. This positive effect may be attributed to the flexibility of eHealth interventions, enabling them to support increased adherence to lifestyle programs and fit in with students’ lifestyles. These positive results are further supported by other advantages of eHealth, including its wide appeal, accessibility, ability to reach a variety of demographics, and high compliance at a cheap cost.

Regarding lipid profile, there was a statistically significant decrease in (TC (< 0.001, < 0.001), LDL (< 0.001, < 0.001), HDL (0.04, 0.005), and RBS (< 0.001, < 0.001) post-intervention compared to pre-intervention in mHealth group and traditional group respectively. Also, in comparing the mHealth group versus the traditional group, there was a statistically significant improvement in the mHealth than the traditional group in TC and LDL (p-value 0.04, 0.05) respectively. The effectiveness of ongoing counseling via mHealth may be responsible for the notable decrease in cholesterol. Communication between patients and healthcare professionals was made possible via mHealth, which improved intervention compliance and gave people access to health information.

Our results are consistent with earlier studies showing that technology-based treatments are more successful than conventional advice at encouraging adherence to healthy lifestyle choices^46,50^. Specifically, when this paradigm was used for cardiometabolic risk management, cholesterol levels improved^51^. Furthermore, two research investigated the efficacy of nutrition-only platforms. One study examined the effects of a computerized meal planning and nutritional tracking platform on lipid markers in people with dyslipidemia and found improvements in all parameters that were tested^52^.

Furthermore, one study investigated the impact of a mobile application that offered health education and tracked step counts on various cardiovascular risk factors within a presumably healthy population^53^. The findings indicated that increased daily step counts associated with app usage led to a reduction of 0.07 mmol/L in LDL cholesterol and a rise of 0.05 mmol/L in HDL cholesterol^53^. By improving lipid profiles, this study contributes to the scant data currently available demonstrating the efficacy of digital lifestyle interventions in lowering cardiovascular risk and correcting hormonal imbalances. These results demonstrate the potential of such interventions for primary and secondary prevention of cardiometabolic risk, in conjunction with previous clinical population-focused investigations.

Compared to a recent meta-analysis, our results showed no significant differences in triglycerides (P = 0.72), total cholesterol (P = 0.44), LDL cholesterol (P = 0.35), or HDL cholesterol (P = 0.21). Nonetheless, positive results were noted in the mHealth groups^54,55^. A meta-analysis by Gencer et al.^56^ demonstrated that a reduction of 1 mmol/L in LDL cholesterol yields significant benefits. Ettehad et al.'s meta-analysis^57^ similarly suggests that a 10 mmHg decrease in SBP correlates with a lowered risk of cardiovascular events. Turan Kavradim et al.^58^ further bolstered this idea by pointing out that improvements were seen in both SBP and DBP. Our results are consistent with those of Akinosun et al. regarding lipid metrics^59^ who observed enhancements in LDL, HDL, and total cholesterol levels. However, Xu et al.^60^ considered that only HDL and total cholesterol levels showed improvement, however, LDL cholesterol did not alter significantly. Furthermore, in contrast to our findings, Turan Kavradim et al.^58^ reported improvements in triglycerides and total cholesterol but not in LDL or HDL cholesterol. Despite this, a meta-analysis found no evidence of a significant advantage of smartphone technology over traditional medical methods in terms of blood pressure and cholesterol variables. High heterogeneity across the studies measuring total and LDL cholesterol levels, however, may be the cause of this disparity^59,61^.

Yang et al.^62^ from Shandong University’s Health Management Center in China showed that after the intervention, individuals in the health management group had significantly lower levels of low-density lipoprotein, systolic and diastolic blood pressure, waist circumference, and BMI. High-density lipoprotein levels, on the other hand, significantly rose, and these indices were lower than those of the control group. Following a 2-year follow-up, the control group’s BMI, waist circumference, systolic and diastolic blood pressure, and low-density lipoprotein levels did not alter significantly from baseline.

### Limitations

Many students refused to participate due to long measurement tools and invasive laboratory investigations and post-session electronic responses collection represented a load on the researcher due to the large number of participants and different methods of receiving it. The study design didn’t support the assessment of long-term sustainability.

## Conclusions

In conclusion, the mHealth intervention program has proven to be a promising and effective avenue for promoting positive lifestyle changes. Its impact on awareness and behavior change suggests that integrating mobile technology into health interventions can be a key strategy for advancing public health and well-being. Refinement of strategies and addressing challenges will be essential for maximizing the potential of mHealth interventions in the future. Further research on different populations should be applied to confirm the effectiveness of the mHealth on the no-medical field students and the general population, in addition to the application of mHealth on other health problems to confirm its role in health promotion. Longitudinal follow up studies on the mHealth program on a large scale are recommended to assess the sustainability of the mHealth program on lifestyle.

## Data Availability

The datasets generated during and/or analyzed during the current study are available from the corresponding author on reasonable request.
